# On a new commensal species of *Aliaporcellana* from the western Pacific (Crustacea, Decapoda, Porcellanidae)

**DOI:** 10.3897/zookeys.780.26388

**Published:** 2018-08-08

**Authors:** Alexandra Hiller, Bernd Werding

**Affiliations:** 1 Smithsonian Tropical Research Institute, Apartado 0843–03092, Panamá, República de Panamá Smithsonian Tropical Research Institute Panama Panama; 2 Institut für Tierökologie und Spezielle Zoologie der Justus-Liebig-Universität Giessen, Heinrich-Buff-Ring 29 (Tierhaus), D-35392 Giessen, Germany Justus-Liebig-Universität Giessen Giessen Germany

**Keywords:** Crustacea, Porcellanidae, *
Aliaporcellana
*, new species, Indo-West Pacific, commensalism, adaptation, sponge- and octocoral-dwelling

## Abstract

*Aliaporcellanaspongicola***sp. n.** from the Philippines and Indonesia is described. The new species has been frequently photographed by divers because of its striking coloration, but has not been described yet. *Aliaporcellanaspongicola***sp. n.** is in fact a widespread commensal of barrel sponges of the genus *Xestospongia* and other sponges. Morphological characters and ecological information of all described species of *Aliaporcellana*, and of other porcellanids associated with sponges and soft corals, suggest that all members of the genus are commensals, and that similar morphological adaptations to dwelling on these hosts have evolved independently in different evolutionary lines within Porcellanidae.

## Introduction

The porcellanid genus *Aliaporcellana* was established by Nakasone and Miyake (1969) for a group of Indo-West Pacific species previously assigned to *Porcellana* Lamarck and to one of three natural groups within *Polyonyx* Stimpson, designated by Johnson (1958) as the *P.denticulatus* Paul’son 1875, group. A diagnostic character considered by Nakasone and Miyake (1969) to raise *Aliaporcellana* is the dactylus of all walking legs bearing two or more distinctively well-developed fixed spines. *Aliaporcellana* contained nine species until Haig (1978) restricted the genus to the species of the *Polyonyxdenticulatus* group, which now includes the type *A.suluensis* (Dana 1852), *A.pygmaea* (de Man 1902) and *A.telestophila* (Johnson 1958), and the species described by Nakasone and Miyake (1969), *A.kikuchii*. A fifth species, *A.taiwanensis*, was subsequently described by Dong et al. (2011).

Here we describe a new sponge-dwelling species of *Aliaporcellana* from material collected in the Philippines and Indonesia. Despite having been frequently photographed by divers because of its striking coloration and relatively large size, the species has not been described. With the exception of *A.telestophila* , commensalism has never been reported for other congeners. We highlight the characters distinguishing the new species from its congeners, and discuss the morphological traits, present in all *Aliaporcellana* species and other porcellanids associated with sponges, which we interpret as adaptations to living on these hosts.

## Material and methods

We found the new species in material collected in the Philippines by G. Paulay [Florida Museum of Natural History, Gainesville, U.S.A. (UF)] and in Indonesia by C.H.J.M. Fransen [Naturalis Leiden, The Netherlands (RMNH)]. The holotype is deposited in the National Museum of Natural History, Philippines (NMCR). Color photographs of the holotype and of the live crab in the field were provided by G. Paulay, and were included in the description. Measurements of carapace length and width (in mm) of type individuals follow collection information.

## Results

### Systematic account

#### Family Porcellanidae

##### Aliaporcellana spongicola sp. n.

Taxon classificationAnimaliaDecapodaPorcellanidae

http://zoobank.org/21BCF647-FA9C-43C6-B604-5FCBD474643D

**Figure 1.**
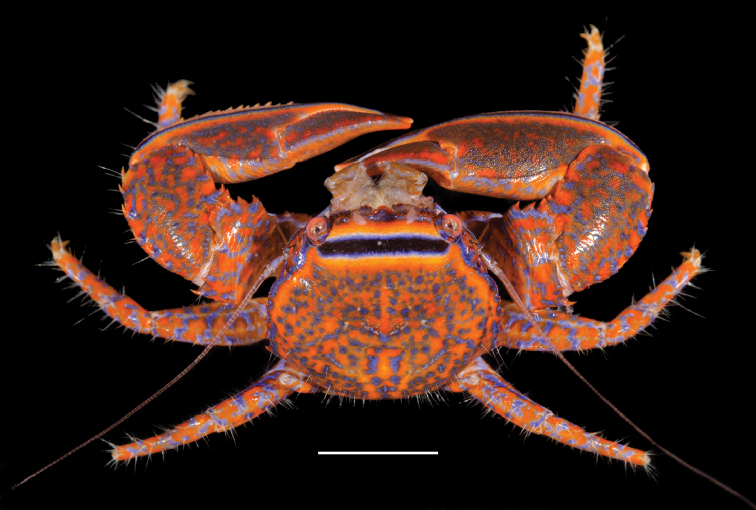
*Aliaporcellanaspongicola* sp. n. Female (ovigerous) paratype, UF 43328 (Photo UF dPHIL 7104), Philippines, Oriental Mindoro Province, Mindoro, Puerto Galera, off Pt W of Bayanar Beach. Scale bar: 3.5 mm.

**Figure 2.**
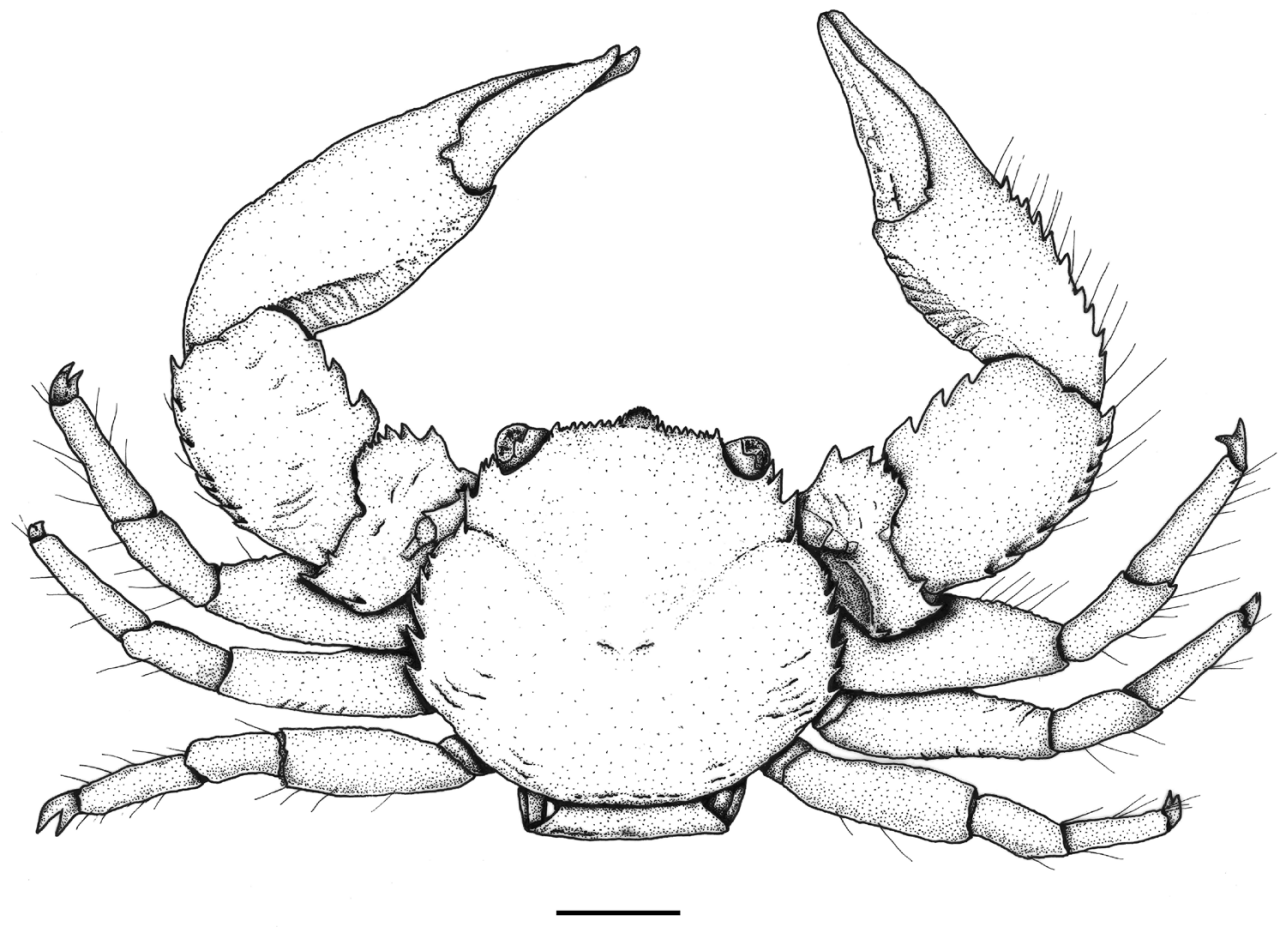
*Aliaporcellanaspongicola* sp. n. Female (ovigerous) holotype, UF 43328, Philippines, Oriental Mindoro Province, Mindoro, Puerto Galera, off Pt W of Bayanar Beach. Scale bar: 2 mm.

**Figure 3.**
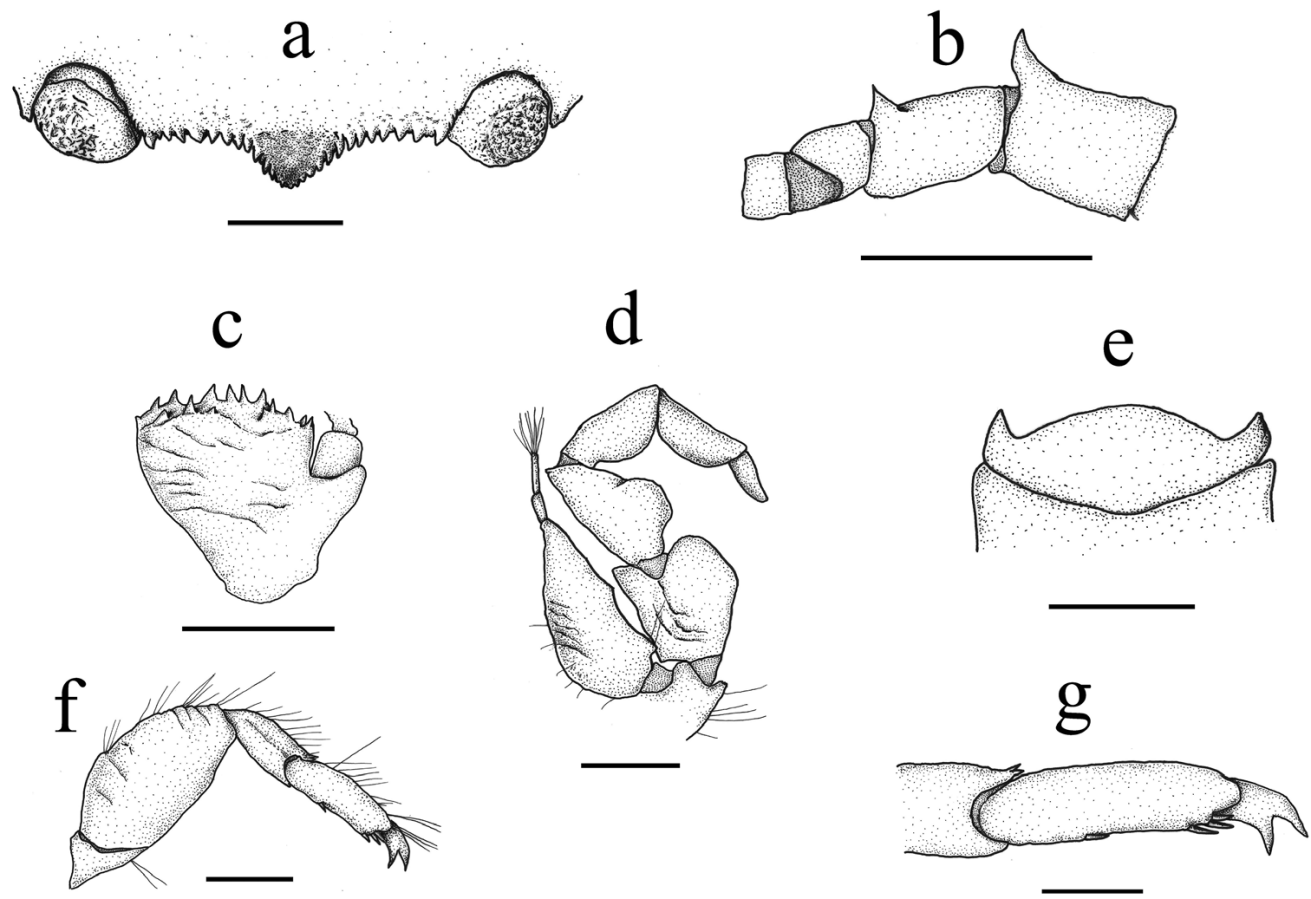
Details of *Aliaporcellanaspongicola* sp. n., female (ovigerous) paratype, UF 43328, Philippines, Oriental Mindoro Province, Mindoro, Puerto Galera, off Pt W of Bayanar Beach. **a** Carapace front **b** Dorsal view of left antennae, movable articulations **c** Dorsal view of left antennular peduncle **d** Dorsal view of left third maxilliped **e** Third thoracic sternite **f** Dorsal view of first right walking leg **g** Detailed view of dactylus of first right walking leg. Scale bars: 1 mm (**a–e, g**); 2 mm (**f**).

**Figure 4.**
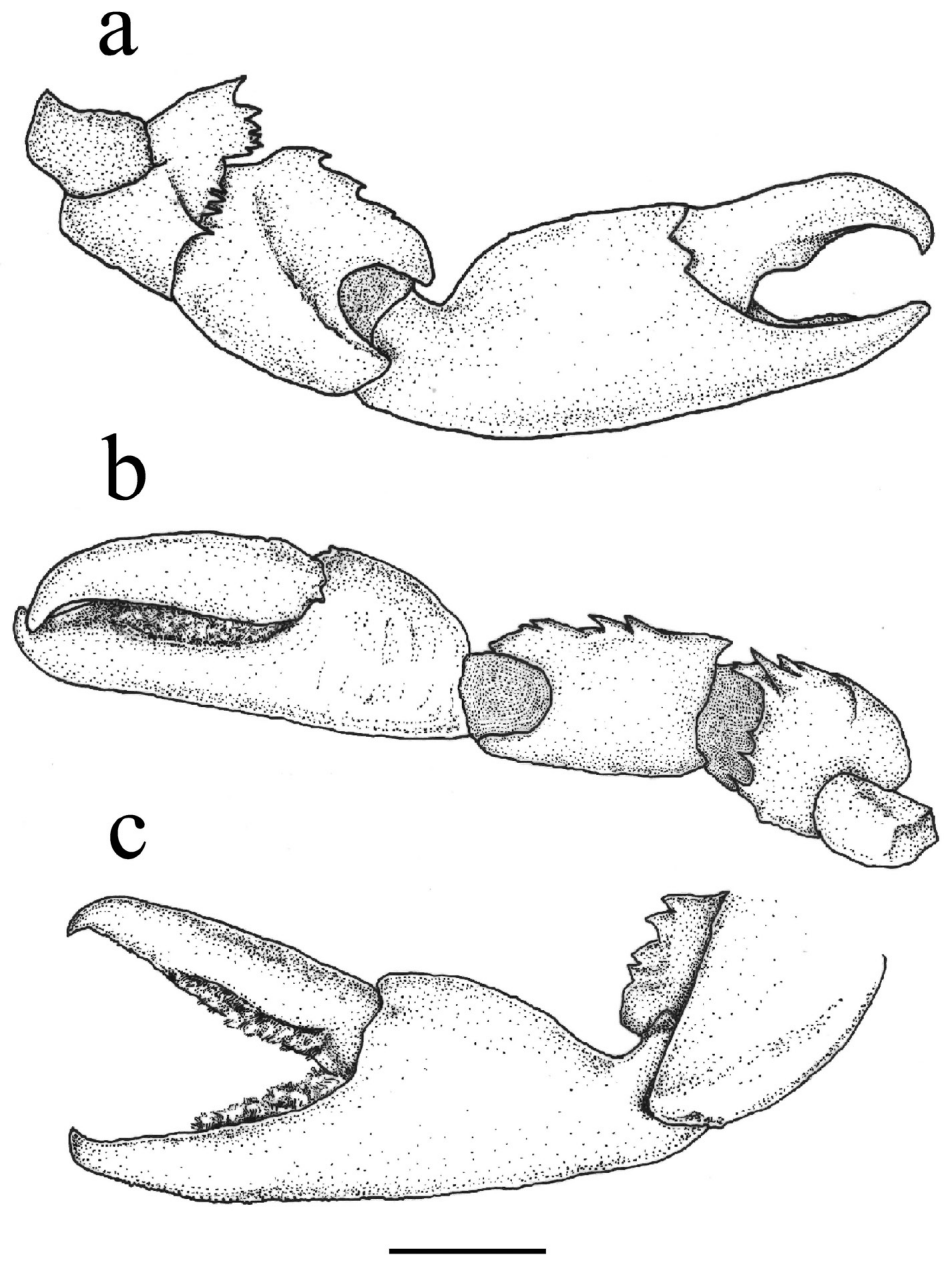
Chelipeds of *Aliaporcellanaspongicola* sp. n., female (ovigerous) holotype, UF 43328, Philippines, Oriental Mindoro Province, Mindoro, Puerto Galera, off Pt W of Bayanar Beach. Ventral view of **a** left cheliped **b** right cheliped **c** manus of right cheliped. Scale bar: 2 mm.

**Figure 5.**
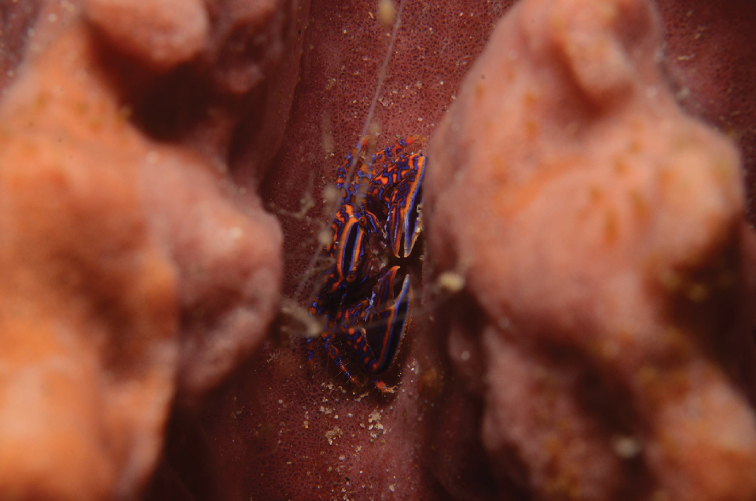
*Aliaporcellanaspongicola* sp. n. Live specimen sitting on barrel sponge (photograph UF dPHIL 09927). Same collection data as holotype.

Figures 1 , 2 , 3 , 4 , 5

###### Material.

**Holotype**: female (ovigerous), NMCR 4966, ex UF 43328, Philippines, Oriental Mindoro Province, Mindoro, Puerto Galera, off Pt W of Bayanar Beach, 13.5118°N 120.9088°E, 10–13 m, sand slope, coll. G. Paulay, 02.04.2015, 6.8 × 7.0 mm. **Paratypes**: 2 females (ovigerous), UF 43328, same collection data as holotype, 7.4 × 7.6 mm, 5.2 × 5.2 mm;1 female (ovigerous), UF 42943, Philippines, Oriental Mindoro Province, Mindoro, Puerto Galera, Batangas Channel, 13.5199°N 120.9604°E, 11 m, lagoon sand slope with sponge, coll. G. Paulay, 12.04.2015, 6.2 × 6.8mm; 2 males, 1 female (ovigerous), RMNH.CRUS.D.57287, Indonesia, SW Sulawesi, Spermonde Archipelago, Bitung, sta. 17, 20 m, from large grey folious sponge, cleaning station, coll. C.H.J.M. Fransen, 30.10.1994, 4.8 × 4.4 mm, 3.3 × 3.0 mm, 5.2 × 4.8 mm.

###### Description.

Carapace rounded (Figures 1, 2), considerably variable in form and in length-width ratio; larger females with carapace broader than long (ratio < 1), smaller individuals with carapace relatively longer than broad (ratio > 1); dorsal surface convex, glossy, with faint, transverse striae on branchial and intestinal regions; cervical grooves gently depressed. Front (Figures 1, 2) broad, slightly produced beyond eyes, weakly trilobate, somewhat deflexed; frontal lobe visible in dorsal view, grooved, overreaching lateral ones. Distal margin of entire front lined with row of rounded, upwardly directed small spines (Figure 3a), the largest on supraocular edges. Outer orbital angles (Figure 2) forming acute, bifid tooth followed by hepatic spine of similar size. Epibranchial margin rounded, produced outwards, marked with epibranchial spine; cervical groove faintly marked. Mesial branchial margins crested, with row of 5 or 6 strong, anteriorly, upwardly directed spines of increasing size posteriorly. Sidewalls entire.

Eyes moderately large (Figures 1, 2, 3a), retracted, ocular peduncles short. First movable segment of antennal peduncle (Figures 2, 3b) with strong, anteriorly curved distal spine, second with smaller, anterodistal, acute protuberance, third one globular. Basal segment of antennular peduncle (Figure 3c) with anterior surface transversely rugose, surrounded with open ring of strong, conical spines. Third maxilliped (Figure 3d) slightly rugose, ischium sub-quadrate with inner lobe, inner margin of merus semi-circular; exopodite long, pyriform, reaching 2/3 of length of merus.

Third thoracic sternite (Figure 3e) broadly elliptic, with triangular, forwardly produced, lateral projections. Telson with 7 plates.

Chelae moderately different in size and form (Figures 2, 4a-c); merus short, dorsal surface faintly rugose, inner margin with strongly projecting, sub-rectangular projection, fringed distally with cockscomb-shaped row of teeth, other large spines on proximal and distal edge of outer margin, one on distal margin; ventral side with two large spines on distal margin. Carpus 1.5 times as long as wide, dorsal surface evenly convex, similarly structured as carapace, with some faint transversal plications; inner margin with 3–5 low or sharply hooked teeth, decreasing in size distally, distal edge rounded. Outer margin with a row of six or seven acute, upwardly directed spines, the last one forming distal edge. Palm slender, surface rounded, similarly structured as carpus, with faint, transverse striae. Smaller chela with outer margin bearing row of approximately ten sharp spines on proximal half, with scattered, long, simple setae; fingers reaching up to half length of chela, dactylus moderately twisted, opening vertically, cutting edges denticulate, without teeth, both fingers with narrow fringe of fine, plumose setae in proximal 2/3 of length. Larger chela somewhat stouter, outer margin with row of spines less developed or disappearing in large specimens, with scattered, long, simple setae, fingers relatively shorter as in smaller chela; dactylus moderately twisted, opening vertically, cutting edges in pollex and dactylus with broad, shallow tooth, gape naked.

Walking legs (Figures 2, 3f, g) stout, merus with some transversal striae, with scattered, long, simple setae, increasing in number towards dactylus; carpus in first and second leg ending dorsodistally in two minute spines, propodus ventrally with 1 movable spine in addition to terminal triplet; dactylus terminating in bifurcate, curved claw.

*Coloration.* The background color of carapace and extremities is bright orange (hexadecimal color #e86700), overlain with a reticulate bright blue (hexadecimal color #000de8) pattern (Figures 1, 5). A broad, black band crosses the carapace transversely at the level of the hepatic region; it is fringed on both sides by a small, blue line and a broad, orange band. A similar band extends along the outer border of the chelipeds from the carpus to the tip of the pollex. In a number of individuals the blue color prevails over the orange, and the entire crab appears blue.

###### Ecology.

*Aliaporcellana* currently consists of six species. Of all species, *A.spongicola* sp. n. is by far the most strikingly colorful, and has, therefore, become popular among underwater photographers and marine aquarists. *Aliaporcellanaspongicola* sp. n. dwells on large barrel sponges of the genus *Xestospongia* Laubenfels [family Petrosiidae; e.g., *X.testudinaria* (Lamarck 1815)] and on other types of sponges, like the “large, grey foliose sponge”, on which the crabs from Sulawesi included in this study, were found. The porcellanid lies in the sponge’s folds, where it is most protected from predators (Figure 5).

###### Distribution.

The type specimens come from the central Philippines and northern Sulawesi, Indonesia.

###### Etymology.

The name *spongicola* (from the Latin word *spongia*, meaning sponge, and the Latin suffix *cola*, meaning dwelling) refers to the sponge-dwelling habit of the new species.

###### Remarks.

*Aliaporcellanaspongicola* sp. n. is considerably variable in the shape of carapace and the degree of spination on body and extremities. As in other porcellanid species, the spines are more defined in smaller specimens. The new species is distinguished from *A.pygmaea* and *A.kikuchii* by the lack of acute spines on the dactylus of the smaller cheliped (Osawa 2007; Dong et al. 2011), and by its smoother surface of carapace and chelipeds (Lewinsohn 1969; Nakasone and Miyake 1969; Werding and Hiller 2007; Osawa and Chan 2010). *Aliaporcellanaspongicola* sp. n. can be distinguished from *A.suluensis*, *A.telestophila* and *A.taiwanensis* by its regularly denticulated front (Figures 2, 3a), which is smooth in the other species, and by the basis of the antennular peduncle, which is crowned with a ring of spines (Figure 3c) and is at most granulate or faintly serrate in the compared species (see Lewinsohn 1969; Werding and Hiller 2007; Dong et al. 2011 for *A.suluensis*; Ng and Goh 1969 for *A.telestophila*; Dong et al. 2011 for *A.taiwanensis*).

## Discussion

With the description of *Aliaporcellanaspongicola* sp. n., the genus now comprises six species.

Up to now, *A.telestophila* is the only species of the genus reported to live as commensal (Johnson 1958; Ng and Goh 1996). Johnson (1958) described this species based on his own collections and observations, highlighting that *A.telestophila* was found “strictly [in] commensalism with the octocoral *Telesto*”. However, Ng and Goh (1996) doubted the identification of the octocoral host and referred to it as *Solenocaulon* Gray (family Anthothelidae Broch), instead. Ng and Goh (1996) and Goh et al. (1999) described the porcellanid as dweller inside of the hollow branches of the octocoral, communicating with the outer medium through the openings of these branches. The species lives in male-female pairs; sometimes two pairs are found in one host colony.

Our own observations of the morphology and ecology of *A.suluensis* collected from sponges in Saudi Arabia, and of all other *Aliaporcellana* species, led us to conclude that perhaps all species of the genus are commensals. We base our conclusions on the well-developed, fixed spines on the dactylus of the walking legs, a character present in all *Aliaporcellana* species (see Figures 3f-g) and other porcellanid commensals that inhabit sponges (e.g., *Pachychelesackleianus* A. Milne-Edwards, 1880, *Polyonyxhendersoni* Southwell, 1909 and *P.splendidus* Sankolli, 1963; see Haig 1960; Hiller et al. 2010). This morphological trait is probably an adaptation to moving on the surface of this type of host. We hypothesize that all members of the genus *Aliaporcellana* are commensal of sponges or octocorals, and that this morphological trait has evolved independently in different evolutionary lines within Porcellanidae. *Aliaporcellanaspongicola* sp. n. probably lives in male-female pairs, as *A.telestophila* does on the octocoral *Solenocaulon* (Ng and Goh 1996; Goh et al. 1999).

The association between crab and sponge may be easily overlooked because sponges are often attached to each other and to rocks, and are damaged when the rocks are lifted. More collection data of other *Aliaporcellana* species are needed to confirm the commensal status of the genus.

## Supplementary Material

XML Treatment for Aliaporcellana spongicola
